# Clinical efficacy of casirivimab and imdevimab in preventing COVID-19 in the Omicron BA.5 subvariant epidemic: a retrospective study

**DOI:** 10.1186/s40780-025-00501-x

**Published:** 2025-10-27

**Authors:** Mariko Ohtani, Takuya Yokoo, Taito Miyazaki, Hiroshi Yasuda, Eriko Nishikawa, Manabu Tomida, Mayumi Tsukada, Emi Sato, Shinobu Hirayama, Hinako Murakami, Sadako Yoshizawa, Takahiro Matsumoto, Kazuhiro Tateda

**Affiliations:** 1 Department of Pharmacy, Toho University Omori Medical Center, 6-11-1 Omori-Nishi, Ota-Ku, Tokyo, 143-8541 Japan; 2 Department of Infection Control and Prevention, Toho University Omori Medical Center, 6-11-1 Omori-Nishi, Ota-Ku, Tokyo, 143-8541 Japan; 3 General Medicine and Emergency Center, Toho University Omori Medical Center, 6-11-1 Omori-Nishi, Ota-Ku, Tokyo, 143-8541 Japan; 4 Department of Nursing, Toho University Omori Medical Center, 6-11-1 Omori-Nishi, Ota-Ku, Tokyo, 143-8541 Japan; 5 Department of Clinical Laboratory, Toho University Omori Medical Center, 6-11-1 Omori-Nishi, Ota-Ku, Tokyo, 143-8541 Japan; 6 Department of Microbiology and Infectious Diseases, Toho University School of Medicine, 5-21-16 Omori-Nishi, Ota-Ku, Tokyo, 143-8540 Japan

**Keywords:** COVID-19, Casirivimab, Imdevimab, Post-exposure prophylaxis, Omicron

## Abstract

**Background:**

The neutralizing monoclonal antibody combination of casirivimab and imdevimab (CAS + IMD) is the only therapy approved for preventing coronavirus disease 2019 (COVID-19) following exposure to severe acute respiratory syndrome coronavirus 2. However, the efficacy of CAS + IMD against Omicron variants remains uncertain, with in vitro studies indicating reduced neutralizing activity. This study aimed to evaluate the clinical efficacy of CAS + IMD in preventing COVID-19 among uninfected hospitalized contacts of patients with COVID-19.

**Methods:**

A retrospective chart review was conducted on 154 inpatients exposed to patients with COVID-19 between October and December 2022. Fifty-two uninfected participants who were unvaccinated or immunosuppressed and had risk factors for severe COVID-19 were included. The primary endpoint was the COVID-19 incidence rate. Statistical analyses included the chi-square test, Fisher's exact test, and Mann–Whitney U test, as appropriate. Factors associated with COVID-19 incidence (*p* < 0.05) in univariate analysis were included in the multivariate logistic regression. Statistical significance was set at *p* < 0.05.

**Results:**

Among the 52 participants, 14 and 38 were included in the CAS + IMD and non-CAS + IMD groups, respectively. The COVID-19 incidence rate was significantly lower in the CAS + IMD group than in the non-CAS + IMD group (14.3% vs. 52.6%, *p* = 0.013). Multivariate analysis identified CAS + IMD administration as significantly associated with reduced COVID-19 incidence (adjusted odds ratio [OR], 0.121; 95% confidence interval [CI], 0.020–0.710; *p* = 0.019), whereas long-term use of immunosuppressive therapy was associated with increased incidence (adjusted OR, 4.320; 95% CI, 1.090–17.126; *p* = 0.037).

**Conclusions:**

CAS + IMD may be effective for post-exposure prophylaxis of COVID-19 during the Omicron BA.5 subvariant epidemic. However, prudent clinical use should consider the circulating variant profile. Further research is warranted to validate CAS + IMD’s role in COVID-19 post-exposure prophylaxis.

## Background

Coronavirus disease 2019 (COVID-19) rapidly spread worldwide following its initial outbreak in Wuhan, China, in December 2019. Severe acute respiratory syndrome coronavirus 2 (SARS-CoV-2), the causative virus of COVID-19, is an enveloped virus with a large RNA genome of approximately 30,000 nucleotides. Since late 2020, numerous SARS-CoV-2 variants with genetic mutations have emerged [1]. Following the emergence of the B.1.1.529 (Omicron) variant in late 2021, successive Omicron subvariants have replaced earlier epidemic strains, exhibiting immune evasion mechanisms, such as increased transmissibility, vaccine resistance, and reduced susceptibility to neutralizing monoclonal antibodies.

Although antiviral agents are frequently used to treat COVID-19, particularly Omicron lineages, none are indicated for prophylaxis. In contrast, in Japan, the only approved post-exposure prophylactic therapy is the neutralizing monoclonal antibody combination of casirivimab and imdevimab (CAS + IMD). A randomized, double-blind, placebo-controlled phase III trial assessed the efficacy of subcutaneous CAS + IMD in preventing SARS-CoV-2 infection among previously uninfected household contacts of infected individuals [2]. During the 28-day assessment, symptomatic SARS-CoV-2 infection rates were 1.5% (11/753) and 7.8% (59/752) in the CAS + IMD and placebo groups, respectively, yielding a relative risk reduction of 81.4% (odds ratio [OR], 0.17; 95% confidence interval [CI], 0.09–0.33; *p* < 0.001). A 7-month follow-up analysis confirmed a sustained prophylactic effect, with an 81.2% reduction in symptomatic SARS-CoV-2 infection compared to placebo (OR, 0.17; 95% CI, 0.10–0.27; *p* < 0.0001), demonstrating both pre- and post-exposure efficacy [3]. However, these findings preceded the emergence of Omicron variants, and although CAS + IMD has shown reduced neutralizing activity against Omicron variants in vitro [4–9], its current effectiveness in preventing COVID-19 remains unclear.

This study aimed to evaluate the clinical efficacy of CAS + IMD in preventing COVID-19 in uninfected hospitalized contacts of patients with COVID-19 during the Omicron BA.5 subvariant epidemic.

## Methods

### Patients

A retrospective chart review was conducted at Toho University Omori Medical Center for inpatients who had contact with patients with COVID-19 between October and December 2022. Contacts were defined as patients admitted to the same hospital room as patients with COVID-19 within the 2 days preceding symptom onset. Those who remained hospitalized underwent a 7-day isolation period following their last exposure. Patients who had been discharged before the discovery of contact were followed up by telephone. To evaluate the clinical efficacy of CAS + IMD, we included uninfected patients who were unvaccinated or immunosuppressed and had risk factors for severe COVID-19, in accordance with the drug’s package insert. Patients were excluded if they were discharged during the isolation period and had no post-discharge clinical information available in their electronic medical records. Additionally, patients with asymptomatic SARS-CoV-2 infection at the time of CAS + IMD administration or who experienced treatment interruption due to adverse effects were excluded to ensure accurate assessment of prophylactic efficacy. The infection control team recommended CAS + IMD to eligible patients upon confirmation of contact with patients with COVID-19. The final decision regarding administration was made by the attending physician. CAS + IMD was administered as a single 1,200-mg dose (600 mg each of CAS and IMD). Nasopharyngeal samples were collected and tested for SARS-CoV-2 using either reverse transcription–polymerase chain reaction (Xpert Xpress SARS-CoV-2; Beckman Coulter, Tokyo, Japan) or antigen testing (Lumipulse SARS-CoV-2 Ag; Fujirebio, Tokyo, Japan) at the time of contact identification and between days 5 and 7 following exposure. Patients who developed symptoms suggestive of COVID-19 before the end of the isolation period were tested as clinically indicated.

### Research methods

Using electronic medical records, we assessed the following characteristics during the study period: age, sex, height, weight, body mass index (BMI), estimated glomerular filtration rate (eGFR), CAS + IMD administration, dialysis, number of vaccinations, performance status, duration of contact with patients with COVID-19, isolation methods, and risk factors for severe COVID-19. These risk factors included age ≥ 50 years, obesity (BMI > 30 kg/m^2^), cardiovascular disease (including hypertension), chronic lung disease (including asthma), diabetes, chronic kidney disease (eGFR < 60 mL/min/1.73 m^2^), and immunosuppressed status, based on the inclusion criteria of a phase III trial that assessed the efficacy of CAS + IMD in outpatients with COVID-19 [10]. Immunosuppressed status encompassed malignancy treatment, bone marrow or organ transplant, end-stage renal failure (eGFR < 15 mL/min/1.73 m^2^ or dialysis), and long-term use of immunosuppressive therapy. Long-term use of immunosuppressive therapy was defined as treatment with prednisolone equivalent to ≥ 20 mg/day for at least two weeks or the use of moderate to severe immunosuppressive agents, such as cyclosporine, tacrolimus, mycophenolate mofetil, or rituximab [11]. However, anticancer agents were classified under malignancy treatment and were not considered part of long-term immunosuppressive therapy. The primary endpoint was the COVID-19 incidence rate.

### Statistical analyses

Participant characteristics were compared using the chi-square test, Fisher's exact test, or the Mann–Whitney U test, as appropriate. COVID-19 incidence rates were compared using the chi-square test. Factors significantly associated with COVID-19 incidence in a univariate analysis (*p* < 0.05) were included as covariates in a multivariate logistic regression analysis. If multicollinearity was detected (Pearson’s correlation coefficient > 0.7), one of the correlated factors was removed prior to model fitting. COVID-19 incidence rates between the CAS + IMD and non-CAS + IMD groups were analyzed using the Kaplan–Meier method and compared with the log-rank test. Data are presented as medians and interquartile ranges (IQR). Statistical significance was set at *p* < 0.05. Analyses were performed using SPSS version 24 (IBM Corp., Armonk, NY, USA).

## Results

### Patients’ characteristics

**Fig. 1:**
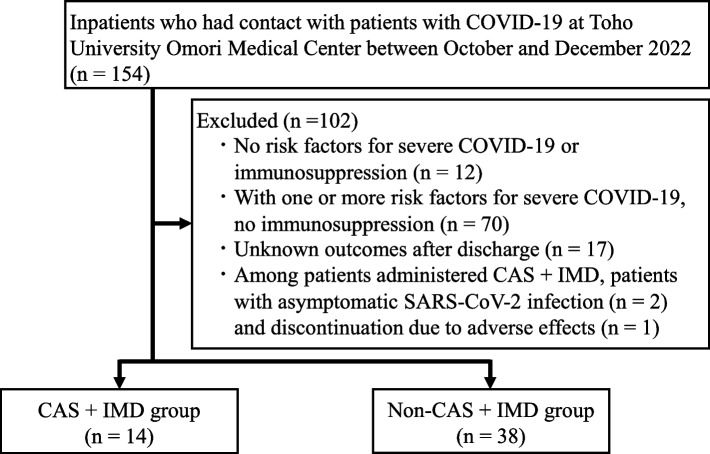
Participant selection flowchart. COVID-19, coronavirus disease 2019; CAS + IMD, casirivimab and imdevimab; SARS-CoV-2, severe acute respiratory syndrome coronavirus 2

Of the 154 inpatients who had contact with patients with COVID-19 during the study period, 102 were excluded for the following reasons: 12 had no risk factors for severe COVID-19 or immunosuppression, 70 had ≥ 1 risk factor for severe COVID-19 but no immunosuppression, 17 had unknown outcomes after discharge, 2 received CAS + IMD for asymptomatic SARS-CoV-2 infection, and 1 discontinued CAS + IMD due to adverse effects. The only adverse effect observed was in the excluded patient, who experienced neck-to-lumbar pain and nausea during administration, leading to discontinuation at half dose. The patient recovered thereafter. The final analysis included 52 patients: 14 in the CAS + IMD group and 38 in the non-CAS + IMD group (Fig. 1). The CAS + IMD group had significantly more patients on dialysis than the non-CAS + IMD group (7 [50.0%] vs. 3 [7.9%] *p* = 0.002) (Table 1). The duration of contact with patients with COVID-19 was significantly longer in the CAS + IMD group (median, 4 days; IQR, 3–5) than in the non-CAS + IMD group (median, 3 days; IQR, 3–3). No other significant differences in baseline characteristics were found between the groups. Regarding immunosuppressed status—a key risk factor for severe COVID-19—the CAS + IMD group had more patients with end-stage renal failure than the non-CAS + IMD group (7 [50.0%] vs. 5 [13.2%], respectively; *p* = 0.010) (Table2).

**Table 1:** Participant characteristics at baseline

	CAS + IMD (*n* = 14)	Non-CAS + IMD (*n* = 38)	*p* Value
Age (years)	70 [57–78]	71 [60–78]	0.910^a^
Male	8 (57.1)	23 (60.5)	0.825^b^
Height (cm)	156.8 [148.3–164.1]	160.0 [153.0–167.5]	0.460^a^
Body weight (kg)	56.5 [47.7–65.7]	53.4 [45.8–65.8]	0.781^a^
BMI (kg/m^2^)	22.0 [20.3–23.4]	21.6 [18.8–23.7]	0.460^a^
eGFR (mL/min/1.73 m^2^)	15.8 [7.5–75.5]	57.7 [41.4–76.7]	0.149^a^
Dialysis	7 (50.0)	3 (7.9)	0.002^c^
Number of vaccinations			0.851^a^
Unvaccinated	0 (0.0)	1 (2.6)	
Three times or fewer	2 (14.3)	7 (18.4)	
Four times	5 (35.7)	8 (21.1)	
Five times	1 (7.1)	3 (7.9)	
Unknown	6 (42.9)	19 (50.0)	
PS grade			0.221^a^
0	3 (21.4)	19 (50.0)	
1	7 (50.0)	9 (23.7)	
2	1 (7.1)	2 (5.3)	
3	2 (14.3)	4 (10.5)	
4	1 (7.1)	4 (10.5)	
Duration of contact with patients with COVID-19 (days)	4[3–5]	3 [3–3]	0.005^a^
Isolation methods			0.785^a^
Private room isolation	3 (21.4)	9 (23.7)	
Cohort isolation	11 (78.0)	26 (68.4)	
Discharge from hospital	0 (0.0)	3 (7.9)	
Number of risk factors for severe COVID-19	3[3–4]	4[2–4]	0.781^a^

Data are presented as median [interquartile range] or n (%)

*CAS + IMD* casirivimab and imdevimab, *BMI* body mass index, *eGFR* estimated glomerular filtration rate, *PS* performance status, *COVID-19* coronavirus disease 2019

^a^Mann–Whitney U test

^b^Chi-square test

^c^Fisher's exact test

**Table 2:** Risk factors for severe COVID-19 and immunosuppressed status

	CAS + IMD (*n* = 14)	Non-CAS + IMD (*n* = 38)	*p* Value
Risk factors for severe COVID-19			
Age ≥ 50 years	12 (85.7)	34 (89.5)	0.519^a^
Obesity (BMI > 30 kg/m^2^)	0 (0.0)	2 (5.3)	0.530^a^
Cardiovascular disease (including hypertension)	11 (78.6)	24 (63.2)	0.240^a^
Chronic lung disease (including asthma)	3 (21.4)	8 (21.1)	0.625^a^
Diabetes	3 (21.4)	11 (28.9)	0.435^a^
Chronic kidney disease (eGFR < 60 mL/min/1.73 m^2^)	10 (71.4)	22 (57.9)	0.374^b^
Others	2 (14.3)	0 (0.0)	0.069^a^
Immunosuppressed status			
Malignancy treatment	2 (14.3)	14 (36.8)	0.108^a^
Bone marrow or organ transplant	2 (14.3)	4 (10.5)	0.519^a^
End-stage renal failure (eGFR < 15 mL/min/1.73 m^2^ or dialysis)	7 (50.0)	5 (13.2)	0.010^a^
Long-term use of immunosuppressive therapy	6 (42.9)	18 (47.4)	0.772^b^
Others	1 (7.1)	1 (2.6)	0.470^a^

Values are presented as n (%)

*COVID-19* coronavirus disease 2019, *CAS + IMD* casirivimab and imdevimab, *BMI* body mass index, *eGFR* estimated glomerular filtration rate

^a^Fisher's exact test

^b^Chi-square test

### Clinical efficacy of CAS + IMD in preventing COVID-19

**Fig. 2:**
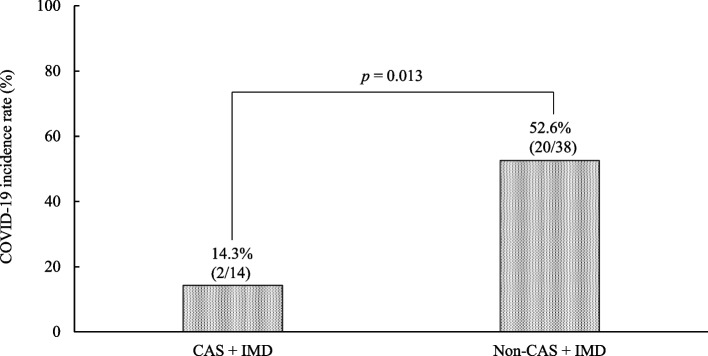
Incidence rate of COVID-19 in each group. COVID-19, coronavirus disease 2019; CAS + IMD, casirivimab and imdevimab

The COVID-19 incidence rates were 14.3% (2/14) in the CAS + IMD group and 52.6% (20/38) in the non-CAS + IMD group. The incidence was significantly lower in the CAS + IMD group (*p* = 0.013) (Fig. 2).

### Factors associated with COVID-19 incidence

**Fig. 3:**
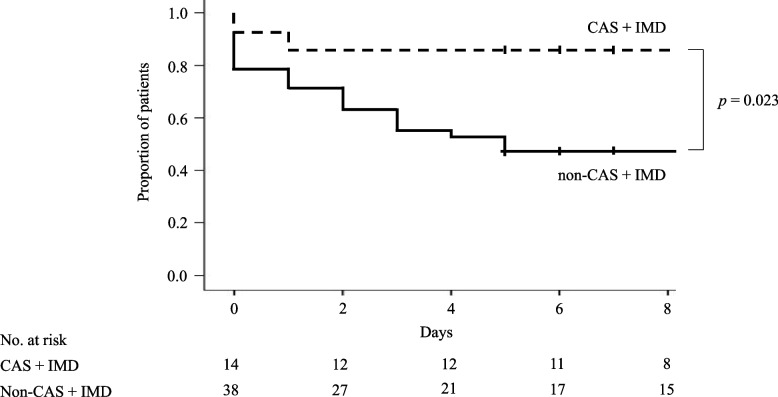
Changes over time in the proportion of patients without COVID-19. COVID-19, coronavirus disease 2019; CAS + IMD, casirivimab and imdevimab

Among the 52 eligible patients, 22 developed COVID-19, and 30 did not (Table 3). Univariate analyses indicated that patients in the COVID-19 group were considerably less likely to have received CAS + IMD and more likely to have undergone bone marrow or organ transplant or long-term immunosuppressive therapy than those in the non-COVID-19 group. Regarding isolation methods, fewer patients in the COVID-19 group were isolated in individual rooms (2 [9.1%] vs. 10 [33.3%], respectively; *p* = 0.010). Since no multicollinearity was observed between these factors, three covariates were selected in order of decreasing *p*-value, considering the number of objective variables—long-term immunosuppressive therapy, CAS + IMD administration, and isolation method—and included in the multivariate logistic regression analysis. The results identified CAS + IMD administration (adjusted OR, 0.121; 95% CI, 0.020–0.710; *p* = 0.019) as a significant protective factor against COVID-19 and long-term use of immunosuppressive therapy (adjusted OR, 4.320; 95% CI, 1.090–17.126; *p* = 0.037) as a significant risk factor. The proportion of patients without COVID-19 was significantly lower in the CAS + IMD group than in the non-CAS + IMD group (*p* = 0.023) (Fig. 3).

**Table 3:** Factors associated with COVID-19 incidence

Variables	Univariate analysis			Multivariate analysis		
	COVID-19 (*n* = 22)	Non-COVID-19 (*n* = 30)	*p* Value	Adjusted odds ratio	95% Confidence interval	*p* Value
Age (years)	70 [58–76]	73 [59–78]	0.711			
Male	14 (63.6)	17 (56.7)	0.613			
Height (cm)	160.2 [153.0–169.6]	157.4 [152.1–163.8]	0.421			
Body weight (kg)	57.3 [46.5–68.0]	53.4 [46.0–64.2]	0.482			
BMI (kg/m^2^)	22.0 [21.3–23.3]	21.4 [19.3–23.9]	0.566			
eGFR (mL/min/1.73 m^2^)	52.5 [43.2–65.3]	55.2 [14.7–87.0]	0.985			
CAS + IMD administration	2 (9.1)	12 (40.0)	0.013	0.121	0.020–0.710	0.019
Dialysis	2 (9.1)	8 (26.7)	0.107			
Number of vaccinations			0.186			
Unvaccinated	0 (0.0)	1 (3.3)				
Three times or fewer	4 (18.2)	5 (16.7)				
Four times	8 (36.4)	5 (16.7)				
Five times	3 (13.6)	1 (3.3)				
Unknown	7 (31.8)	18 (60.0)				
PS grade			0.617			
0	11 (50.0)	11 (36.7)				
1	4 (18.2)	12 (40.0)				
2	3 (13.6)	0 (0.0)				
3	2 (9.1)	4 (13.3)				
4	2 (9.1)	3 (10.0)				
Duration of contact with patients with COVID-19 (days)	3 [3–3]	3 [3–4]	0.367			
Isolation methods			0.010	4.914	0.910–26.522	0.064
Private room isolation	2 (9.1)	10 (33.3)				
Cohort isolation	17 (77.3)	20 (66.7)				
Discharge from hospital	3 (13.6)	0 (0.0)				
Risk factors for severe COVID-19						
Age ≥ 50 years	19 (86.4)	27 (90.0)	0.506			
Obesity (BMI > 30 kg/m^2^)	2 (9.1)	0 (0.0)	0.174			
Cardiovascular disease (including hypertension)	16 (72.7)	19 (63.3)	0.476			
Chronic lung disease (including asthma)	5 (22.7)	6 (20.0)	0.538			
Diabetes	8 (36.4)	6 (20.0)	0.189			
Chronic kidney disease (eGFR < 60 mL/min/1.73 m^2^)	15 (68.2)	17 (56.7)	0.399			
Others	0 (0.0)	2 (6.7)	0.328			
Immunosuppressed status						
Malignancy treatment	5 (22.7)	11 (36.7)	0.282			
Bone marrow or organ transplant	5 (22.7)	1 (3.3)	0.042			
End-stage renal failure (eGFR < 15 mL/min/1.73 m^2^ or dialysis)	3 (13.6)	9 (30.0)	0.166			
Long-term use of immunosuppressive therapy	15 (68.2)	9 (30.0)	0.006	4.320	1.090–17.126	0.037
Others	0 (0.0)	2 (6.7)	0.328			

Data are presented as median [interquartile range] or n (%)

*COVID-19* coronavirus disease 2019, *BMI* body mass index, *eGFR* estimated glomerular filtration rate, *CAS + IMD* casirivimab and imdevimab, *PS* performance status

## Discussion

In this study, we evaluated the clinical efficacy of CAS + IMD in preventing COVID-19 among unvaccinated or immunosuppressed, uninfected inpatients with risk factors for severe COVID-19 who had been in contact with patients with COVID-19. The results demonstrated a markedly lower COVID-19 incidence rate in the CAS + IMD group than in the non-CAS + IMD group. Multivariate analysis identified CAS + IMD administration and long-term immunosuppressive therapy as remarkable factors associated with COVID-19 incidence. This study is novel in evaluating the clinical efficacy of CAS + IMD for post-exposure prophylaxis during the Omicron BA.5 subvariant epidemic, a context that has not yet been validated in clinical trials. Accordingly, the findings may provide valuable clinical insights.

A phase III trial assessing the preventive efficacy of CAS + IMD was conducted prior to the Omicron variant epidemic [2]. In contrast, the present study was conducted during the Omicron variant epidemic, involving different predominant strains. Although this study did not perform genetic sequencing of SARS-CoV-2 strains, national surveillance data from Japan during the study period indicated that BA.5 accounted for an average of 92.1% of infections, followed by BA.2 (6.2%) and BA.4 (0.2%) [12]. Therefore, BA.5 was likely the dominant strain during the study period.

CAS + IMD targets distinct, non-overlapping epitopes on the receptor-binding domain (RBD) of the SARS-CoV-2 spike protein, thereby inhibiting viral entry into host cells [13]. Omicron variants possess multiple RBD mutations and various antigenic deletions and substitutions in the amino-terminal domain, enabling them to evade most therapeutic monoclonal antibodies [1]. Takashita et al. evaluated in vitro neutralizing activity and reported that CAS + IMD was 317.8-fold less effective against BA.5 than against the ancestral strain and 43.0–143.6-fold less effective against BA.2 and other non-Omicron variants [4, 5]. Conversely, a retrospective study found no significant difference in the therapeutic efficacy of CAS + IMD between the Delta and Omicron variants. However, that study was conducted before the BA.5 subvariant became prevalent [14, 15]. In the present study, conducted during the BA.5 subvariant epidemic, the CAS + IMD group showed considerably lower COVID-19 incidence than the non-CAS + IMD group (Fig. 2), and CAS + IMD use was associated with reduced incidence (Table 3). These findings suggest that reduced neutralizing activity in vitro may not directly translate to diminished clinical efficacy. Hagihara et al. reported on patients with hematological malignancies and persistent SARS-CoV-2 infection despite prior antiviral treatment (remdesivir or molnupiravir) during the BA.5 epidemic. CAS + IMD was administered, and viral RNA became undetectable within 7 days in 5 of 9 patients, despite isolated viruses showing low or no sensitivity to CAS + IMD [16]. One proposed explanation is that antibody-dependent cellular cytotoxicity (ADCC) activity against Omicron variants may have contributed to viral clearance. Wang et al. performed immunoprofiling in 46 participants from a single site in a multisite CAS + IMD trial using longitudinal blood samples collected before the emergence of Delta and Omicron variants and prior to widespread vaccination [17]. Compared with placebo, CAS + IMD administration accelerated the transition from an acute inflammatory immunophenotype to a resolution phase characterized by reduced tissue injury, lower proinflammatory markers, and restoration of lymphocyte–monocyte balance, regardless of baseline serostatus. CAS + IMD also preserved host T-cell immunity to the SARS-CoV-2 spike protein. The observed ADCC activity and anti-inflammatory properties of CAS + IMD may help explain its role in reducing COVID-19 incidence, even amid BA.5 predominance.

Timely detection of COVID-19 cases, isolation of patients, and identification and isolation of contacts are essential to prevent outbreaks in hospitals and nursing homes. Although private room isolation is preferable due to the potential for transmission among contacts, only 23.1% (12/52) of participants in this study were isolated in private rooms, whereas 71.2% (37/52) were isolated in shared rooms. This likely reflects the challenge of implementing ideal isolation protocols during cluster outbreaks across multiple wards. The COVID-19 group had a markedly lower rate of private room isolation than the non-COVID-19 group in the univariate analysis (Table 3). Notably, despite considerably longer exposure to patients with COVID-19, the CAS + IMD group experienced markedly lower COVID-19 incidence than the non-CAS + IMD group (Table 1, Fig. 2).

Many hospitalized patients are immunosuppressed and at elevated risk for severe COVID-19, and infection may delay treatment for underlying conditions. In this study, long-term immunosuppressive therapy was identified as a factor increasing COVID-19 incidence (Table 3). This study included four patients receiving rituximab for malignancy, all of whom were considered to have severe immunosuppression. Therefore, long-term immunosuppressive therapy in this analysis did not include agents with such potent immunosuppressive effects. In such settings, post-exposure prophylaxis, alongside infection control, becomes especially important to prevent further transmission. Although in vitro studies suggest that CAS + IMD exhibits limited neutralizing activity against Omicron variants post-BA.5 [6, 8, 9], its ADCC activity and SARS-CoV-2–neutralizing anti-inflammatory effects may still contribute to reduced incidence. Therefore, its use should be carefully considered based on the prevalent variant. In Japan, all monoclonal antibodies for SARS-CoV-2 were previously government-distributed and free of charge, but distribution ended on May 31, 2024. Currently, there are virtually no agents available for post-exposure prophylaxis, despite recurring COVID-19 outbreaks. As with influenza, a strategic stockpile of agents for post-exposure prophylaxis is essential for COVID-19 preparedness.

This study has some limitations, including its single-center, retrospective design, small sample size, and lack of randomization, which may introduce bias. Vaccination is the basis of COVID-19 prevention. However, since simultaneous COVID-19 cases occurred in the hospital, it was not possible to confirm the vaccination status of all patients during the study period, and the vaccination status of 48.1% (25/52) or approximately half of the patients in this study was unknown. Even among the 70 patients who were excluded because they had ≥ 1 risk factor for severe COVID-19 but no immunosuppression, 40 (57.1%) were excluded because their vaccination status could not be confirmed, and the majority were older adults (median, 72 years; IQR, 63–78). However, the first vaccination coverage in Japan is reported to be 80.3% for individuals of all ages and 94.4% for those aged 65 years and older [18]; consequently, the proportion of unvaccinated individuals among these 40 excluded patients is assumed to be small. Therefore, the number of excluded patients is unlikely to have biased the results of this study. On the other hand, even in patients whose vaccination status could be confirmed, the period since the last vaccination was not yet documented and could not be fully evaluated, which may have affected the evaluation of the clinical efficacy of CAS + IMD. Additionally, we did not assess the presence of anti-SARS-CoV-2 antibodies; therefore, it was not possible to evaluate whether seroreactivity from prior vaccination influenced the clinical efficacy of CAS + IMD. Furthermore, due to the COVID-19 epidemic, some of the cases identified as concentrated contacts on day 0 may have actually been true index cases who were not properly interviewed or examined for symptoms at the time. This misclassification may have influenced the decision to administer CAS + IMD and its observed efficacy. The considerably higher number of dialysis patients in the CAS + IMD group may reflect selection bias, potentially due to greater willingness among renal center physicians to administer the agent. Although these findings suggest CAS + IMD is effective for post-exposure prophylaxis during the Omicron BA.5 subvariant epidemic, the current absence of suitable prophylactic agents poses a critical challenge. With emerging variants that may evade immunity, these results aim to inform future research on effective post-exposure prophylactic strategies.

## Conclusions

The findings of this study suggest that CAS + IMD is effective for post-exposure prophylaxis of COVID-19 during the Omicron BA.5 subvariant epidemic. However, prudent decision-making should consider the prevalence of circulating variants. Further research is warranted to establish optimal strategies for post-exposure prophylaxis of COVID-19.

## Data Availability

The datasets used and/or analyzed during the current study are available from the corresponding author on reasonable request.

## Abbreviations

ADCC: Antibody-dependent cellular cytotoxicity
CAS: Casirivimab
CI: Confidence interval
COVID-19: Coronavirus disease 2019
IMD: Imdevimab
IQR: Interquartile range
OR: Odds ratio
RBD: Receptor-binding domain
SARS-CoV-2: Severe acute respiratory syndrome coronavirus 2
